# Cardiovascular disease protein biomarkers are associated with kidney function: The Framingham Heart Study

**DOI:** 10.1371/journal.pone.0268293

**Published:** 2022-05-11

**Authors:** Amena Keshawarz, Shih-Jen Hwang, Gha Young Lee, Zhi Yu, Chen Yao, Anna Köttgen, Daniel Levy

**Affiliations:** 1 Population Sciences Branch, Division of Intramural Research, National Heart, Lung, and Blood Institute, National Institutes of Health, Bethesda, Maryland, United States of America; 2 Framingham Heart Study, Framingham, Massachusetts, United States of America; 3 Broad Institute of Harvard and the Massachusetts Institute of Technology, Cambridge, Massachusetts, United States of America; 4 Department of Epidemiology, Johns Hopkins Bloomberg School of Public Health, Baltimore, Maryland, United States of America; 5 Welch Center for Prevention, Epidemiology, and Clinical Research, Johns Hopkins University, Baltimore, Maryland, United States of America; 6 Institute of Genetic Epidemiology, Faculty of Medicine and Medical Center, University of Freiburg, Freiburg, Germany; University of Liège, BELGIUM

## Abstract

**Background:**

Biomarkers common to chronic kidney disease (CKD) and cardiovascular disease (CVD) may reflect early impairments underlying both diseases.

**Methods:**

We evaluated associations of 71 CVD-related plasma proteins measured in 2,873 Framingham Heart Study (FHS) Offspring cohort participants with cross-sectional continuous eGFR and with longitudinal change in eGFR from baseline to follow-up (ΔeGFR). We also evaluated the associations of the 71 CVD proteins with the following dichotomous secondary outcomes: prevalent CKD stage ≥3 (cross-sectional), new-onset CKD stage ≥3 (longitudinal), and rapid decline in eGFR (longitudinal). Proteins significantly associated with eGFR and ΔeGFR were subsequently validated in 3,951 FHS Third Generation cohort participants and were tested using Mendelian randomization (MR) analysis to infer putatively causal relations between plasma protein biomarkers and kidney function.

**Results:**

In cross-sectional analysis, 37 protein biomarkers were significantly associated with eGFR at FDR<0.05 in the FHS Offspring cohort and 20 of these validated in the FHS Third Generation cohort at p<0.05/37. In longitudinal analysis, 27 protein biomarkers were significantly associated with ΔeGFR at FDR<0.05 and 12 of these were validated in the FHS Third Generation cohort at p<0.05/27. Additionally, 35 protein biomarkers were significantly associated with prevalent CKD, five were significantly associated with new-onset CKD, and 17 were significantly associated with rapid decline in eGFR. MR suggested putatively causal relations of melanoma cell adhesion molecule (MCAM; -0.011±0.003 mL/min/1.73m^2^, p = 5.11E-5) and epidermal growth factor-containing fibulin-like extracellular matrix protein 1 (EFEMP1; -0.006±0.002 mL/min/1.73m^2^, p = 0.0001) concentration with eGFR.

**Discussion/conclusions:**

Eight protein biomarkers were consistently associated with eGFR in cross-sectional and longitudinal analysis in both cohorts and may capture early kidney impairment; others were implicated in association and causal inference analyses. A subset of CVD protein biomarkers may contribute causally to the pathogenesis of kidney impairment and should be studied as targets for CKD treatment and early prevention.

## Introduction

Chronic kidney disease (CKD) affects approximately 15% of the United States population, including more than 30% of adults over the age of 65 years [1, 2]. Additionally, CKD is among the leading global causes of mortality [3]. CKD is characterized by kidney damage and impairment of filtration, which can culminate in kidney failure and death [4]. Kidney function is frequently assessed clinically using estimated glomerular filtration rate (eGFR) based on serum creatinine concentration [5]. Early stages of CKD are often asymptomatic, and as such, CKD may not be diagnosed until the development of substantial and often irreversible kidney dysfunction. Thus, the identification of biomarkers of subclinical CKD that detect early kidney impairment when treatment is more likely to be beneficial could prove to be important for disease prevention and treatment.

CKD and cardiovascular disease (CVD) share common risk factors including diabetes mellitus (DM) and hypertension [6], which are highly prevalent in adults with CKD and are associated with subclinical and clinical CVD risk [7, 8]. More than 50% of CKD cases in the United States are attributable to DM [9], and estimates of hypertension prevalence in patients with CKD range between 40% and 60% [10–12]. Other shared mechanisms contributing to CKD and CVD include inflammation [13], activation of the renin-angiotensin-aldosterone system [13], oxidative stress [14, 15], and endothelial dysfunction [14]. Identifying biomarkers of kidney function may provide insights into shared mechanisms of CKD and CVD.

Given the complex relationship between CKD and CVD, we aimed to identify protein biomarkers of kidney dysfunction and CKD. To this end, we evaluated the associations of 71 CVD-related plasma proteins with CKD traits. These proteins were measured in 7,184 Framingham Heart Study (FHS) participants as a part of the Systems Approach to Biomarker Research in Cardiovascular Disease (SABRe CVD) Initiative [16]. We tested the cross-sectional and longitudinal associations of the 71 CVD proteins with kidney function traits. We also conducted causal inference analyses using genetic variants associated with these proteins in conjunction with genetic variants from a recently published large GWAS meta-analysis of kidney function traits [17].

## Materials and methods

### Discovery sample

The discovery sample for this investigation included participants in the FHS Offspring [18] cohort who attended the seventh examination cycle (1998–2001; baseline visit). At this visit, serum and plasma samples were collected for measurement of serum creatinine and the 71 CVD-related plasma proteins as part of the SABRe CVD Initiative [16]. Participants with a baseline eGFR ≥ 60 mL/min/1.73m^2^ who attended the FHS Offspring eighth examination cycle (2005–2008; follow-up visit) where a second serum creatinine measurement was obtained were included in the longitudinal analysis of kidney function.

**Fig 1:**
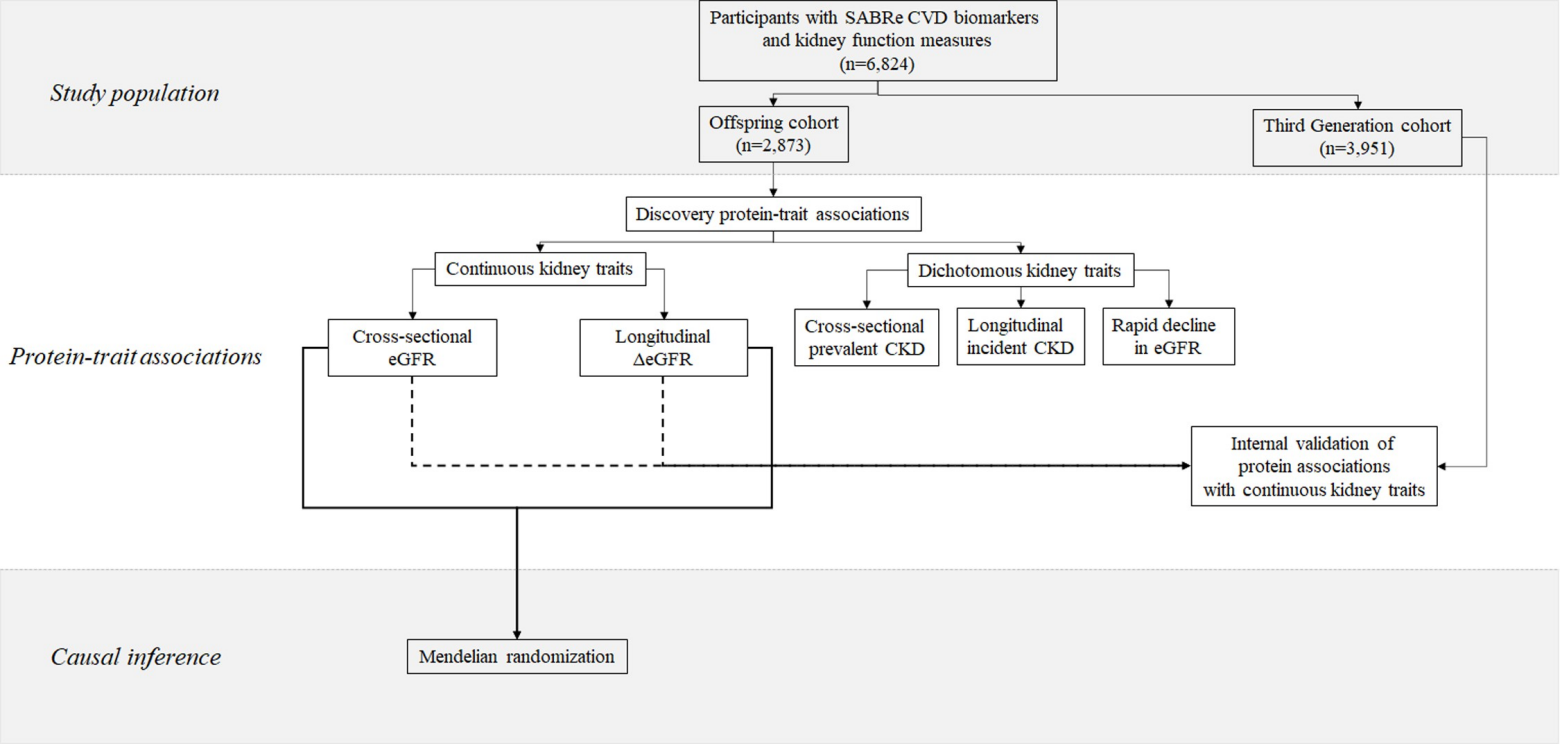
Overview of the study design.

Participants with a medical record-confirmed diagnosis of heart failure or myocardial infarction at the baseline visit were excluded due to the effect of these diagnoses on biomarker concentrations, yielding a final sample of 2,873 participants for cross-sectional analysis. There were 2,393 participants who attended the follow-up examination and were eligible for inclusion in the longitudinal analyses. All study protocols were approved by the Boston University Medical Center institutional review board, and all study participants provided their informed consent to participate in FHS research investigations. Data used in this study can be accessed through the National Center for Biotechnology Information Database of Genotypes and Phenotypes (accession number, phs00007.v29.p10). An overview of the study design is presented in **Fig 1.**

### Clinical examination and definitions

Medical history and fasting blood samples were collected from all study participants at an in-person research center examination [19]. Body mass index (BMI) was calculated as the ratio of the participant’s weight in kilograms to the square of height in meters. DM status was defined by fasting blood glucose concentration ≥126 mg/dL or use of hypoglycemic medication [20]. Hypertension was defined by systolic blood pressure ≥ 140 mmHg, diastolic blood pressure ≥ 90 mmHg, or use of antihypertensive medication [21]. Participants were defined as current smokers if they reported smoking at least one cigarette per day on average during the previous year. Prevalent CVD at the baseline study visit was defined as angina pectoris, coronary insufficiency, cerebrovascular accident, atherothrombotic infarction of brain, transient ischemic attack, cerebral embolism, intracerebral hemorrhage, subarachnoid hemorrhage, or intermittent claudication.

### SABRe CVD biomarkers

Details of measurement of the 71 CVD-related proteins evaluated in this study have been described previously [16]. Briefly, plasma samples were obtained from all participants at their baseline clinical examinations and stored at -80°C. Samples were assayed and concentrations of 85 biomarkers were quantified using a modified ELISA sandwich approach on a Luminex xMAP platform (Sigma-Aldrich, St. Louis, MO); 71 of these had detectable levels in at least 95% of participant samples and were used in the present analyses. The 71 protein biomarkers used in this study and their molecular weights are presented in **S1 Table**. Due to the distribution of protein concentration values, inverse-rank normalized protein concentration values were used in all statistical analyses.

### Kidney function traits

Serum creatinine was measured using the modified Jaffe method (Roche Diagnostics, Indianapolis, IN) and calibrated to National Health and Nutrition Examination Survey (NHANES) III creatinine values [6]. eGFR, an estimate of kidney function, was calculated using the CKD Epidemiology Collaboration (CKD-EPI) creatinine equation [5]. The annual change in eGFR (ΔeGFR) was calculated for each participant as the follow-up minus baseline eGFR value divided by the number of years between the two visits.

This study primarily focused on the continuous eGFR and ΔeGFR measures of kidney function, with a secondary emphasis on dichotomous traits of CKD and rapid decline in eGFR. Participants were categorized as having CKD stage ≥ 3 at the baseline visit if they had eGFR values <60 mL/min/1.73m^2^. New-onset CKD stage ≥ 3 was defined as a follow-up eGFR <60 mL/min/1.73m^2^ in participants who had eGFR values ≥60 mL/min/1.73m^2^ at baseline and a concomitant ≥30% reduction in eGFR between baseline and follow-up [22]. Rapid decline in eGFR was defined as a decline in eGFR of at least 3 mL/min/1.73m^2^ per year between baseline and follow-up.

### Meta-analyzed GWAS of kidney function traits

Publicly available kidney trait GWAS summary statistics were obtained from the CKDGen Consortium website (https://ckdgen.imbi.uni-freiburg.de/) as reported in a 2019 meta-analysis that identified genetic variants associated with eGFR and CKD (eGFR < 60 mL/min/1.73m^2^) in trans-ethnic and European Ancestry populations [17].

### Association of protein biomarkers with kidney traits

The primary kidney function outcomes were cross-sectional eGFR at the baseline visit, and longitudinal ΔeGFR (i.e., eGFR at follow-up minus eGFR at baseline). We generated log-transformed eGFR outcome values and applied the transformed values for subsequent regression analysis. The statistical associations of each protein biomarker with these traits were evaluated using linear mixed models with a random intercept.

The secondary kidney function outcomes evaluated were cross-sectional CKD at the baseline visit, new-onset CKD at follow-up, and longitudinal rapid decline in eGFR. The statistical associations between variation in the protein distribution and these dichotomous kidney function traits were evaluated using logistic regression models.

All multivariable models were adjusted for the following kidney dysfunction risk factors at baseline: age, sex, BMI, systolic blood pressure, total and HDL cholesterol, blood pressure and/or lipid medication use, prevalent CVD, DM, and cigarette smoking status (current smokers vs. non-smokers). Longitudinal analyses (i.e., models evaluating ΔeGFR, new-onset CKD, and rapid decline in eGFR) were additionally adjusted for baseline eGFR, and only those participants with available longitudinal data and who had a baseline eGFR ≥ 60 mL/min/1.73m^2^ were included in the model assessing new-onset CKD (n = 2,257). We defined a “clinical model” including all covariates except the protein biomarker of interest (i.e., reduced model), and a “full model” including all covariates in the clinical model and the protein biomarker of interest.

For each protein biomarker, we evaluated the quantitative contribution of the protein biomarker to variation in continuous kidney trait outcomes by comparing the goodness-of-fit R^2^ value of the main and full models. The change in R^2^ (ΔR^2^) illustrates the incremental impact of the addition of the protein biomarker over and above the clinical model. For the dichotomous kidney function traits, we compared receiver operating characteristic (ROC) curves between the clinical and full models using a contrast matrix to quantify the difference in the area under the empirical ROC curve (i.e., the c-statistic). Results of the ROC curve comparison fit a chi-square distribution, which was used to evaluate statistical significance. We calculated the false discovery rate (FDR) for all associations, and FDR values <0.05 were interpreted as statistically significant. Discovery protein-trait statistical analyses were performed in SAS version 9.4 (SAS Institute, Cary, NC).

### Validation of continuous kidney function outcomes

We conducted internal validation of the protein biomarkers significantly associated with eGFR in cross-sectional discovery analysis and with ΔeGFR in longitudinal discovery analyses. Protein biomarkers that were significantly associated with eGFR in the FHS Offspring cohort were subsequently validated in 3,951 participants in the FHS Third Generation cohort [23] using data from the first (2002–2005) and second (2009–2011) exams of this cohort. Third Generation cohort participants were included in the internal validation sample if they had serum creatinine and the SABRe plasma protein measurements and did not have a medical record-confirmed diagnosis of heart failure or myocardial infarction. Statistical significance of the internal validation analyses was defined as a Bonferroni-corrected p-value of 0.05/total number of proteins included in the validation analysis. Validation analyses were performed in SAS version 9.4 (SAS Institute, Cary, NC).

### Two-sample Mendelian randomization (MR)

We used a two-sample MR approach [24] to test the hypothesis that protein concentrations associated with eGFR in the discovery and validation protein-trait analyses are causally related to continuous eGFR using data from published kidney trait GWAS [17]. Prior genome-wide association studies (GWAS) of SABRe proteins in FHS participants identified protein quantitative trait loci (pQTL) variants for 57 of the 71 protein biomarkers [25]. Of these previously identified variants, we considered only *cis-*pQTL variants for protein biomarkers that were significantly associated with cross-sectional eGFR or longitudinal ΔeGFR in the validation analysis in the Third Generation cohort. *cis-*pQTL variants were defined as those single nucleotide polymorphisms (SNPs) that were located 1 megabase upstream or downstream of the protein-coding gene’s transcription start site. Furthermore, *cis*-pQTL variants were pruned at linkage disequilibrium r^2^ < 0.01, and only those that overlapped with SNPs from the kidney function GWAS were used as instrumental variables for the protein biomarkers.

For protein biomarkers with only one pQTL variant as an instrumental variant in MR analysis after pruning, the causal effect was calculated using the Wald test. For protein biomarkers with more than one pQTL variant after pruning, the causal effect was calculated using inverse-variance weighted meta-analyzed estimates. MR results were interpreted as statistically significant after applying a Bonferroni correction for the total number of unique protein biomarkers tested across all outcomes (i.e., p<0.05/n, where n is the number of protein-trait associations tested).

In the case of multiple *cis*-pQTL variants contributing to a significant weighted causal estimate, we conducted sensitivity analyses for heterogeneity. When more than two pQTL variants or SNPs contributed to a significant weighted causal estimate, we additionally tested for horizontal pleiotropy and evaluated the change in the effect estimate after excluding one variant at a time from the calculation (i.e., leave-one-out analysis).

All MR analyses were conducted in R version 4.0.2 using the *TwoSampleMR* package [24, 26].

## Results

### Participant characteristics

Baseline characteristics of participants in the FHS Offspring and Third Generation cross-sectional cohorts are presented in **Table 1**. The mean age of participants in the Offspring cohort was 60 ± 9 years and mean eGFR was 84 ± 16 mL/min/1.73m^2^. Seven percent of the Offspring cohort had CKD at baseline, while the prevalence of DM and hypertension were 10% and 43%, respectively. The mean age of participants in the Third Generation cohort was 40 ± 9 years and mean eGFR was 102 ± 14 mL/min/1.73m^2^. Less than one percent of the Third Generation cohort sample had CKD, while the prevalence of DM and hypertension were 3% and 18%, respectively.

**Table 1:** Baseline demographic and clinical participant characteristics.

Characteristic	Offspring cohort (n = 2,873)	Third Generation cohort (n = 3,951)
Age, years	60 ± 9	40 ± 9
Women, n (%)	1,573 (55%)	2,109 (53%)
eGFR, mL/min/1.73m^2^	84 ± 16	102 ± 14
CKD, n (%)	210 (7%)	10 (0.3%)
BMI, kg/m^2^	27.95 ± 5.22	26.87 ± 5.55
Fasting glucose, mg/dL	102.99 ± 24.59	95.02 ± 17.9
Systolic blood pressure, mmHg	126.18 ± 18.54	116.69 ± 14.02
Diastolic blood pressure, mmHg	74.15 ± 9.7	75.26 ± 9.63
Total cholesterol, mg/dL	200.64 ± 36.42	188.81 ± 35.46
HDL cholesterol, mg/dL	54.41 ± 17.2	54.41 ± 16.16
DM, n (%)	280 (10%)	114 (3%)
Hypertension, n (%)	1242 (43%)	726 (18%)
Current cigarette smoker, n (%)	362 (13%)	605 (15%)
Cholesterol-lowering medication use, n (%)	564 (20%)	341 (9%)
Prevalent cardiovascular disease, n (%)	308 (11%)	38 (1%)

Abbreviations: eGFR, estimated glomerular filtration rate; CKD, chronic kidney disease; BMI, body mass index; HDL, high density lipoprotein; DM, diabetes mellitus

All continuous characteristics are presented as mean ± standard deviation. All categorical variables are presented as total n (column %) in that category.

Follow-up characteristics of FHS Offspring (discovery) cohort participants included in longitudinal analysis of ΔeGFR, new-onset CKD, and rapid decline in eGFR are presented in **S2 Table**. The mean age of Offspring cohort participants at follow-up was 66 ± 9 years and mean annual ΔeGFR between baseline and follow-up was -0.97 ± 1.73 mL/min/1.73m^2^. Among participants who did not have CKD at baseline, 3% experienced new-onset CKD during follow-up while 91% maintained their eGFR at over 60 mL/min/1.73m^2^. The remaining participants had a follow-up eGFR < 60 mL/min/1.73m^2^, but had a reduction in eGFR < 30%. Eleven percent of the Offspring cohort sample included in longitudinal analysis experienced rapid decline in eGFR between baseline and follow-up.

### Discovery and validation of cross-sectional and longitudinal protein-eGFR associations

Cross-sectional associations of each of the 71 protein biomarkers with eGFR and the incremental increase in model R^2^ associated with each protein biomarker are presented in **S3 Table**. Thirty-seven protein biomarkers were significantly associated with eGFR (at FDR<0.05) in cross-sectional analysis, and of these, all but one (butyrylcholine esterase) had an inverse association with eGFR. Six protein biomarkers were significantly associated with a change in R^2^ ≥ 5%, including alpha-1-microglobulin (A1M), adipsin, beta-2-microglobulin (B2M), cystatin C, myoglobin, and resistin. Of the 37 protein biomarkers that were significantly associated with eGFR in the Offspring cohort in cross-sectional analysis, 20 were also significantly associated with eGFR in the Third Generation cohort at p = 0.05/37 = 0.00135 **(S4 Table)**.

Longitudinal associations of each of the 71 protein biomarkers with ΔeGFR and the incremental increase in model R^2^ associated with each protein biomarker are presented in **S5 Table**. Twenty-seven protein biomarkers were significantly associated with ΔeGFR at FDR<0.05. All significant protein biomarkers except two (plasminogen activator inhibitor 1 [PAI1] and dipeptidyl dipeptidase [DPP4]) were inversely associated with ΔeGFR. B2M and cystatin C were associated with the largest increases in R^2^ (ΔR^2^ = 3.3% and 2.5%, respectively). Of the 27 proteins that were significantly associated with ΔeGFR in the Offspring cohort in longitudinal analysis, 12 were also significantly associated with ΔeGFR in the Third Generation cohort at p = 0.05/27 = 0.00185 (**S6 Table)**.

Biomarkers associated with both eGFR and ΔeGFR in both the Offspring and Third Generation cohorts are presented in **Table 2.** Eight protein biomarkers were consistently associated with continuous kidney function in both cohorts and in both cross-sectional and longitudinal analysis: adipsin, A1M, B2M, cystatin C, epidermal growth factor containing fibulin-like extracellular matrix protein 1 (EFEMP1), myoglobin, soluble receptor for advanced glycation endproducts (sRAGE), and tissue inhibitor of metalloproteinases 1 (TIMP1).

**Table 2:** Biomarkers associated with eGFR and ΔeGFR in the FHS Offspring and Third Generation cohorts.

Protein biomarker	Protein biomarker abbreviation	eGFR, Offspring		eGFR, Third Generation		ΔeGFR, Offspring		ΔeGFR, Third Generation	
		β ± SE	FDR	β ± SE	p-value	β ± SE	FDR	β ± SE	p-value
Adipsin	--	-0.09 ± 0.004	<1E-27	-0.04 ± 0.002	<1E-27	-0.22 ± 0.04	7.3E-07	-0.16 ± 0.03	1.2E-06
Alpha-1-microglobulin	A1M	-0.06 ± 0.004	<1E-27	-0.02 ± 0.002	3.5E-12	-0.18 ± 0.04	1.1E-05	-0.17 ± 0.03	3.6E-08
Beta-2-microglobulin	B2M	-0.10 ± 0.004	<1E-27	-0.03 ± 0.002	<1E-27	-0.43 ± 0.04	9.9E-21	-0.13 ± 0.03	3.7E-05
Cystatin C	--	-0.10 ± 0.004	<1E-27	-0.04 ± 0.003	<1E-27	-0.38 ± 0.04	6.8E-16	-0.21 ± 0.03	1.8E-10
Epidermal growth factor containing fibulin-like extracellular matrix protein 1	EFEMP1	-0.05 ± 0.004	1.7E-26	-0.01 ± 0.003	8.7E-4	-0.17 ± 0.04	2.8E-04	-0.18 ± 0.03	1.6E-07
Myoglobin	--	-0.07 ± 0.004	<1E-27	-0.04 ± 0.003	<1E-27	-0.20 ± 0.04	1.5E-05	-0.13 ± 0.04	3.3E-04
Soluble receptor for advanced glycation endproducts	sRAGE	-0.03 ± 0.004	3.0E-14	-0.01 ± 0.002	6.4E-07	-0.10 ± 0.03	0.02	-0.11 ± 0.03	9.4E-05
Tissue inhibitor of metalloproteinases 1	TIMP1	-0.06 ± 0.004	<1E-27	-0.02 ± 0.002	4.4E-16	-0.21 ± 0.04	8.2E-06	-0.14 ± 0.03	4.5E-06

### Discovery protein-trait associations: Cross-sectional associations with secondary outcomes in the Offspring cohort

Cross-sectional associations of each of the 71 protein biomarkers with prevalent CKD and the associated change in the C-statistic are presented in **S7 Table**. Thirty-five protein biomarkers were significantly associated with prevalent CKD at FDR<0.05, and of these, all but one (angiopoietin-like 3) were associated with greater log-odds of CKD. Ten protein biomarkers were associated with a statistically significant change in the C-statistic.

### Discovery protein-trait associations: Longitudinal associations with secondary outcomes in the Offspring cohort

Five protein biomarkers were significantly associated with higher log-odds of new-onset CKD at FDR<0.05 (**S8 Table)**, while 17 protein biomarkers were significantly associated with higher log-odds of rapid decline in eGFR **(S9 Table)**. No biomarkers were associated with a significant increase in the model C-statistic for either rapid decline in eGFR or new-onset CKD.

### Causal inference

Twenty-four unique proteins were significantly associated with eGFR in both the Offspring and Third Generation in cross-sectional and/or longitudinal analyses; therefore, statistical significance for all MR analyses was set at a Bonferroni-corrected p-value of 0.05/24 = 0.00208. The 24 protein biomarkers associated with eGFR had a total of 25 *cis-*pQTL variants that overlapped with SNPs in the eGFR GWAS [17] and were included in MR analyses. Significant results of MR analysis (protein à kidney trait) for proteins that were significantly associated with eGFR or with ΔeGFR are presented in **Table 3**; full MR results for all protein biomarkers with *cis*-pQTL variants are presented in **S10 Table.** Higher concentrations of epidermal growth factor-containing fibulin-like extracellular matrix protein 1 (EFEMP1) and melanoma cell adhesion molecule (MCAM) were significantly associated with lower eGFR (β ± SE -0.0064 ± 0.0016 and -0.0111 ± 0.0027, respectively), consistent with a potentially causal relationship. Sensitivity analysis of the two EFEMP1 pQTL variants indicated no heterogeneity (p = 0.69, **S11 Table**).

**Table 3:** Causal inference results: MR results for biomarkers significantly associated with eGFR in cross-sectional analyses.

Protein biomarker exposure	Protein biomarker abbreviation	Kidney trait outcome	n_snp_	β ± SE	p-value
Melanoma cell adhesion molecule	MCAM	eGFR (continuous)	1	-0.0111 ± 0.0027	5.11E-5
Epidermal growth factor containing fibulin-like extracellular matrix protein 1	EFEMP1	eGFR (continuous)	2	-0.0064 ± 0.0016	1.04E-4

## Discussion/conclusion

We identified a total of 43 CVD-related protein biomarkers associated with eGFR, a continuous measure of kidney function, in cross-sectional and/or longitudinal discovery analysis. Among these, eight proteins were significantly associated with both eGFR and ΔeGFR in the FHS Offspring discovery sample and the FHS Third Generation validation sample. Of the 37 proteins associated with eGFR and 27 proteins associated with ΔeGFR in the FHS Offspring cohort (i.e. discovery), 20 and 12 were associated with the corresponding trait in the FHS Third Generation cohort (i.e. validation). This group of protein biomarkers included EFEMP1 and MCAM, which were putatively causally related to eGFR in MR analysis. To our knowledge, causal relations of protein biomarkers to CKD based on an integrative genomic approach have not been reported previously.

CKD and CVD share several risk factors; identifying early mechanisms linking the two disease processes may provide insights into targeted interventions to prevent or delay the onset of clinically overt disease. The most notable shared risk factors are DM and hypertension. DM is associated with systemic inflammation and oxidative stress [27], which in turn are associated with microvascular and macrovascular complications and kidney damage [28, 29]. Hypertension results in prolonged exposure of the heart, kidney, and vasculature to elevated hemodynamic load that can contribute to left ventricular hypertrophy, vascular stiffening, heart failure, and CKD [30]. Over-activation of the renin-angiotensin-aldosterone system, which increases blood pressure through multiple mechanisms, can damage glomerular cells [13]. Treatment of hypertension can prevent CKD progression [31], while conversely, kidney disease can lead to resistant hypertension [32]. Furthermore, inflammation also contributes to development of both CKD and CVD. In CKD, the concentration of inflammatory proteins is inversely related to eGFR, and the cytokine interleukin (IL)-6 is a reliable predictor of adverse clinical outcomes in individuals with CKD [14, 33, 34]. Chronic inflammation also may cause oxidative stress and endothelial dysfunction, which contribute to microvascular damage, new-onset or worsening CKD, and clinical CVD [15].

The eGFR values observed in the FHS Offspring participants included in the discovery stage of this study were mostly in the normal range and few individuals experience a rapid decline in eGFR (n = 265; 11%) or progressed to new-onset CKD (n = 72; 3%) during follow-up. Under normal conditions, the glomerulus is able to freely filter proteins with molecular weight (MW) < 70 kilodaltons (kDa), and as such, many of the proteins identified in the cross-sectional and longitudinal protein-trait analyses may reflect early impaired kidney clearance of proteins with low molecular weights with a resultant increase in concentrations of circulating proteins [35]. Indeed, the vast majority of protein biomarkers showed negative associations with eGFR (i.e., lower eGFR was associated with higher protein levels), thus many of the protein biomarkers identified in this study are likely downstream biomarkers of reduced kidney function. Accordingly, the protein biomarkers with consistent and highly significant associations are well-established markers of kidney function, and our validation in the Third Generation cohort of many of the discovery associations observed in the Offspring cohort emphasize associations of these proteins with kidney function in a younger, healthier population with little prevalent CKD. Eight protein biomarkers were associated with both eGFR and ΔeGFR in both the Offspring and Third Generation cohorts, including adipsin, adrenomedullin (ADM), A1M, B2M, collagen type XVIII alpha 1 (COL18A1), cystatin C, EFEMP1, and fibroblast growth factor 23 (FGF23). Of these, cystatin C (MW 15.8 kDa) and B2M (MW 13.7 kDa) were associated with all kidney traits and are used in alternative eGFR calculations due to their low MW and their role as early indicators of kidney impairment, and they are additionally associated with CVD outcomes [16, 36–39]. Other protein biomarkers significantly associated with eGFR and ΔeGFR are also associated with inflammation and kidney disease and may represent non-traditional markers of kidney function due to parallel mechanisms relevant also to CVD. A1M (MW 29.8 kDa), while having a slightly higher MW than either cystatin C or B2M, has also been proposed as a biomarker of kidney disease progression [40–42]. Similarly, adipsin (MW 27.0 kDa), ADM (MW 20.4 kDa), FGF23 (MW 28.0 kDa), and endostatin, a 20 kDa fragment of the 178.2 kDa COL18A1, were all reported to be elevated in the setting of kidney disease in prior studies [43–47]. These protein biomarkers showed a robust association with kidney traits and it is possible that they reflect CKD risk early in the subclinical disease process.

We hypothesized *a priori* that a subset of proteins associated with kidney function may be causal contributors to renal impairment. A recent MR analysis suggested a putatively causal role of melanoma-derived growth regulatory protein (MIA), cystatin M, and carbonic anhydrase III in kidney disease [48]. While these proteins were not included in our assay, our MR analysis evaluated 24 new protein biomarkers and revealed putatively causal inverse relations of EFEMP1 and MCAM with eGFR. EFEMP1 is an extracellular matrix protein involved in both cellular structure and signaling, and it was associated with worse eGFR cross-sectionally and with ΔeGFR longitudinally, with prevalent CKD, and with rapid decline in eGFR. A prior study reported elevated EFEMP1 concentration in human kidney tissue with immunoglobulin A (IgA) nephropathy [49]. The causal mechanism is still unclear, but animal models suggest that EFEMP1 may be associated with vascular remodeling in hypertension [50]. MCAM, also known as MUC18 or CD146, is expressed in endothelial cells and is significantly elevated in individuals with diabetic nephropathy [51, 52] and CKD [53, 54]. Results of our protein-trait analysis similarly showed an association between MCAM with both eGFR and CKD in cross-sectional analyses.

This study has several limitations that must be noted. The primary limitation is that a custom assay used to measure the protein biomarkers in this study, which limited external replication of our results. To address this limitation, we performed validation of the discovery results from the FHS Offspring cohort in participants from the younger and healthier Third Generation cohort in whom CKD was rare (0.3% prevalent CKD at baseline). Independent external replication of the protein biomarkers identified in this study is needed.

In addition, we estimated kidney function based on a single creatinine measurement at both baseline and follow-up, rather than using multiple eGFR values at each time point. As such, our definition of CKD stage ≥ 3 does not mirror the recommended clinical diagnosis of CKD, which requires the presence of kidney structural or functional abnormalities for at least three months [55]. Furthermore, the younger and healthier cohort used for validation analyses limited our ability to identify protein biomarkers of changing kidney function and CKD in longitudinal analysis. Albuminuria and blood urea nitrogen may be clinically useful in the evaluation of patients with impaired kidney function, but these data were not available for analysis in this study. Our study population was limited to adults of European ancestry, which may limit generalizability to other ancestry groups. Functional studies of the proteins reported to have putatively causal relations to kidney function in this investigation may be warranted.

The primary strengths of this study are the large sample size and the integrative genomic approach we employed. We reported cross-sectional and longitudinal protein-trait association results to identify numerous proteins associated with kidney function and its change over time. By integrating pQTL data for the proteins we measured with genetic variants from large GWAS of kidney disease traits we identified putatively causal proteins for kidney function that represent promising candidates for further studies with the ultimate goal to better treat or prevent CKD.

The results of the comprehensive cross-sectional and longitudinal analyses in this study validate proteins that may detect of impaired kidney function early in the disease process when treatment is most likely to be beneficial. We identified robust, significant associations between 20 protein biomarkers with eGFR in cross-sectional discovery and validation analyses and 12 proteins with ΔeGFR in longitudinal analyses both in discovery and validation. Additionally, two proteins were found to be putatively causal for reduced kidney function in causal inference testing. Further studies are necessary to determine if any of the proteins identified by MR can serve as useful biomarkers for CKD either individually or in combination with known and validated biomarkers.

## Supporting information

S1 Table Molecular weights of SABRe CVD protein biomarkers from UniProt database. (XLSX)

S2 Table Follow-up characteristics of Offspring and Third Generation participants included in longitudinal analyses. (XLSX)

S3 Table Adjusted associations between SABRe CVD biomarkers and eGFR in cross-sectional linear regression models in FHS Offspring cohort. (XLSX)

S4 Table Adjusted associations between SABRe CVD biomarkers and eGFR in cross-sectional linear regression models in FHS Third Generation cohort. (XLSX)

S5 Table Adjusted associations between SABRe CVD biomarkers and ΔeGFR in longitudinal linear regression models in FHS Offspring cohort. (XLSX)

S6 Table Adjusted associations between SABRe CVD biomarkers and ΔeGFR in longitudinal linear regression models in FHS Third Generation cohort. (XLSX)

S7 Table Adjusted associations between SABRe CVD biomarkers and CKD in cross-sectional logistic regression models in the FHS Offspring cohort. (XLSX)

S8 Table Adjusted associations between SABRe CVD biomarkers and incident CKD in longitudinal logistic regression models in the FHS Offspring cohort. (XLSX)

S9 Table Adjusted associations between SABRe CVD biomarkers and rapid decline in eGFR in longitudinal logistic regression models in the FHS Offspring cohort. (XLSX)

S10 Table MR results of biomarker pQTLs with continuous eGFR and ΔeGFR. (XLSX)

S11 Table Sensitivity analysis results for MR evaluating EFEMP1 and eGFR. (XLSX)

## References

[1] Saran R, Robinson B, Abbott KC, Agodoa LYC, Bhave N, Bragg-Gresham J, et al. US Renal Data System 2017 Annual Data Report: Epidemiology of Kidney Disease in the United States. Vol. 71, American journal of kidney diseases: the official journal of the National Kidney Foundation. 2018. p. A7. doi: 10.1053/j.ajkd.2018.01.002 29477157 PMC6593155

[2] Joubert BR, Felix JF, Yousefi P, Bakulski KM, Just AC, Breton C, et al. DNA Methylation in Newborns and Maternal Smoking in Pregnancy: Genome-wide Consortium Meta-analysis. Am J Hum Genet [Internet]. 2016;98(4):680–96. Available from: http://www.sciencedirect.com/science/article/pii/S0002929716000707. doi: 10.1016/j.ajhg.2016.02.019 27040690 PMC4833289

[3] Bikbov B, Purcell CA, Levey AS, Smith M, Abdoli A, Abebe M, et al. Global, regional, and national burden of chronic kidney disease, 1990–2017: a systematic analysis for the Global Burden of Disease Study 2017. Lancet [Internet]. 2020 Feb 29;395(10225):709–33. Available from: 10.1016/S0140-6736(20)30045-3 32061315 PMC7049905

[4] Gansevoort RT, Matsushita K, van der Velde M, Astor BC, Woodward M, Levey AS, et al. Lower estimated GFR and higher albuminuria are associated with adverse kidney outcomes. A collaborative meta-analysis of general and high-risk population cohorts. Kidney Int. 2011 Jul;80(1):93–104. doi: 10.1038/ki.2010.531 21289597 PMC3959732

[5] Levey AS, Stevens LA, Schmid CH, Zhang YL, Castro AF 3rd, Feldman HI, et al. A new equation to estimate glomerular filtration rate. Ann Intern Med. 2009 May;150(9):604–12. doi: 10.7326/0003-4819-150-9-200905050-00006 19414839 PMC2763564

[6] Fox CS, Larson MG, Leip EP, Culleton B, Wilson PWF, Levy D. Predictors of New-Onset Kidney Disease in a Community-Based Population. JAMA [Internet]. 2004 Feb 18;291(7):844–50. Available from: 10.1001/jama.291.7.844 14970063

[7] Flint AC, Conell C, Ren X, Banki NM, Chan SL, Rao VA, et al. Effect of Systolic and Diastolic Blood Pressure on Cardiovascular Outcomes. N Engl J Med [Internet]. 2019 Jul 17;381(3):243–51. Available from: 10.1056/NEJMoa1803180 31314968

[8] Dokken BB. The Pathophysiology of Cardiovascular Disease and Diabetes: Beyond Blood Pressure and Lipids. Diabetes Spectr [Internet]. 2008 Jul 1;21(3):160 LP– 165. Available from: http://spectrum.diabetesjournals.org/content/21/3/160.abstract.

[9] Zelnick LR, Weiss NS, Kestenbaum BR, Robinson-Cohen C, Heagerty PJ, Tuttle K, et al. Diabetes and CKD in the United States Population, 2009–2014. Clin J Am Soc Nephrol [Internet]. 2017 Dec 7;12(12):1984 LP– 1990. Available from: http://cjasn.asnjournals.org/content/12/12/1984.abstract. doi: 10.2215/CJN.03700417 29054846 PMC5718269

[10] United States Renal Data System. 2020 USRDS Annual Data Report: Epidemiology of kidney disease in the United States. Bethesda, MD; 2020. 10.1053/j.ajkd.2021.01.002 PMC8148988 33752804

[11] Kibria GM Al, Crispen R. Prevalence and trends of chronic kidney disease and its risk factors among US adults: An analysis of NHANES 2003–18. Prev Med reports [Internet]. 2020 Sep 1;20:101193. Available from: https://pubmed.ncbi.nlm.nih.gov/33101883. doi: 10.1016/j.pmedr.2020.101193 PMC7578737 33101883

[12] Centers for Disease Control and Prevention. Chronic Kidney Disease Surveillance System—United States. [Internet]. 2020. Available from: http://www.cdc.gov/ckd.

[13] Gajjala PR, Sanati M, Jankowski J. Cellular and Molecular Mechanisms of Chronic Kidney Disease with Diabetes Mellitus and Cardiovascular Diseases as Its Comorbidities. Front Immunol [Internet]. 2015 Jul 8;6:340. Available from: https://pubmed.ncbi.nlm.nih.gov/26217336. doi: 10.3389/fimmu.2015.00340 26217336 PMC4495338

[14] Cachofeiro V, Goicochea M, de Vinuesa SG, Oubiña P, Lahera V, Luño J. Oxidative stress and inflammation, a link between chronic kidney disease and cardiovascular disease: New strategies to prevent cardiovascular risk in chronic kidney disease. Kidney Int [Internet]. 2008 Dec 1;74:S4–9. Available from: 10.1038/ki.2008.516. 19034325

[15] Podkowińska A, Formanowicz D. Chronic Kidney Disease as Oxidative Stress- and Inflammatory-Mediated Cardiovascular Disease. Antioxidants (Basel, Switzerland) [Internet]. 2020 Aug 14;9(8):752. Available from: https://pubmed.ncbi.nlm.nih.gov/32823917. doi: 10.3390/antiox9080752 PMC7463588 32823917

[16] Ho JE, Lyass A, Courchesne P, Chen G, Liu C, Yin X, et al. Protein Biomarkers of Cardiovascular Disease and Mortality in the Community. J Am Heart Assoc [Internet]. 2018 Jul 13;7(14):e008108. Available from: https://pubmed.ncbi.nlm.nih.gov/30006491. doi: 10.1161/JAHA.117.008108 30006491 PMC6064847

[17] Wuttke M, Li Y, Li M, Sieber KB, Feitosa MF, Gorski M, et al. A catalog of genetic loci associated with kidney function from analyses of a million individuals. Nat Genet [Internet]. 2019;51(6):957–72. Available from: 10.1038/s41588-019-0407-x 31152163 PMC6698888

[18] Kannel WB, Feinleib M, McNamara PM, Garrison RJ, Castelli WP. An investigation of coronary heart disease in families: the Framinham Offspring Study. Am J Epidemiol [Internet]. 1979 Sep 1;110(3):281–90. Available from: 10.1093/oxfordjournals.aje.a112813 474565

[19] Tsao CW, Vasan RS. Cohort Profile: The Framingham Heart Study (FHS): overview of milestones in cardiovascular epidemiology. Int J Epidemiol [Internet]. 2015 Dec;44(6):1800–13. Available from: https://pubmed.ncbi.nlm.nih.gov/26705418. doi: 10.1093/ije/dyv337 26705418 PMC5156338

[20] Association AD. 2. Classification and Diagnosis of Diabetes: &lt;em&gt;Standards of Medical Care in Diabetes—2020&lt;/em&gt; Diabetes Care [Internet]. 2020 Jan 1;43(Supplement 1):S14 LP-S31. Available from: http://care.diabetesjournals.org/content/43/Supplement_1/S14.abstract. 10.2337/dc20-S002 31862745

[21] Chobanian A V, Bakris GL, Black HR, Cushman WC, Green LA, Izzo JL, et al. Seventh Report of the Joint National Committee on Prevention, Detection, Evaluation, and Treatment of High Blood Pressure. Hypertension [Internet]. 2003 Dec 1;42(6):1206–52. Available from: 10.1161/01.HYP.0000107251.49515.c2 14656957

[22] Levey AS, Inker LA, Matsushita K, Greene T, Willis K, Lewis E, et al. GFR decline as an end point for clinical trials in CKD: a scientific workshop sponsored by the National Kidney Foundation and the US Food and Drug Administration. Am J kidney Dis Off J Natl Kidney Found. 2014 Dec;64(6):821–35. doi: 10.1053/j.ajkd.2014.07.030 25441437

[23] Splansky GL, Corey D, Yang Q, Atwood LD, Cupples LA, Benjamin EJ, et al. The Third Generation Cohort of the National Heart, Lung, and Blood Institute’s Framingham Heart Study: Design, Recruitment, and Initial Examination. Am J Epidemiol [Internet]. 2007 Jun 1;165(11):1328–35. Available from: 10.1093/aje/kwm021 17372189

[24] Hemani G, Zheng J, Elsworth B, Wade KH, Haberland V, Baird D, et al. The MR-Base platform supports systematic causal inference across the human phenome. Elife. 2018 May;7. doi: 10.7554/eLife.34408 29846171 PMC5976434

[25] Yao C, Chen G, Song C, Keefe J, Mendelson M, Huan T, et al. Genome‐wide mapping of plasma protein QTLs identifies putatively causal genes and pathways for cardiovascular disease. Nat Commun [Internet]. 2018;9(1):3268. Available from: 10.1038/s41467-018-05512-x 30111768 PMC6093935

[26] Hemani G, Tilling K, Davey Smith G. Orienting the causal relationship between imprecisely measured traits using GWAS summary data. PLoS Genet. 2017 Nov;13(11):e1007081. doi: 10.1371/journal.pgen.1007081 29149188 PMC5711033

[27] Odegaard AO, Jacobs DRJ, Sanchez OA, Goff DCJ, Reiner AP, Gross MD. Oxidative stress, inflammation, endothelial dysfunction and incidence of type 2 diabetes. Cardiovasc Diabetol. 2016 Mar;15:51. doi: 10.1186/s12933-016-0369-6 27013319 PMC4806507

[28] Lv W, Booz GW, Wang Y, Fan F, Roman RJ. Inflammation and renal fibrosis: Recent developments on key signaling molecules as potential therapeutic targets. Eur J Pharmacol [Internet]. 2017/12/08. 2018 Feb 5;820:65–76. Available from: https://pubmed.ncbi.nlm.nih.gov/29229532. doi: 10.1016/j.ejphar.2017.12.016 29229532 PMC6733417

[29] Jha JC, Ho F, Dan C, Jandeleit-Dahm K. A causal link between oxidative stress and inflammation in cardiovascular and renal complications of diabetes. Clin Sci (Lond). 2018 Aug;132(16):1811–36. doi: 10.1042/CS20171459 30166499

[30] Nwabuo CC, Appiah D, Moreira HT, Vasconcellos HD, Yano Y, Reis JP, et al. Long-term cumulative blood pressure in young adults and incident heart failure, coronary heart disease, stroke, and cardiovascular disease: The CARDIA study. Eur J Prev Cardiol. 2020 Apr;2047487320915342. 10.1177/2047487320915342 PMC8653578 34695218

[31] Ptinopoulou AG, Pikilidou MI, Lasaridis AN. The effect of antihypertensive drugs on chronic kidney disease: a comprehensive review. Hypertens Res [Internet]. 2013;36(2):91–101. Available from: 10.1038/hr.2012.157 23051659

[32] Carey RM, Calhoun DA, Bakris GL, Brook RD, Daugherty SL, Dennison-Himmelfarb CR, et al. Resistant Hypertension: Detection, Evaluation, and Management: A Scientific Statement From the American Heart Association. Hypertens (Dallas, Tex 1979) [Internet]. 2018 Nov;72(5):e53–90. Available from: https://pubmed.ncbi.nlm.nih.gov/30354828. 10.1161/HYP.0000000000000084 PMC6530990 30354828

[33] Sun J, Axelsson J, Machowska A, Heimbürger O, Bárány P, Lindholm B, et al. Biomarkers of Cardiovascular Disease and Mortality Risk in Patients with Advanced CKD. Clin J Am Soc Nephrol [Internet]. 2016/06/08. 2016 Jul 7;11(7):1163–72. Available from: https://pubmed.ncbi.nlm.nih.gov/27281698. doi: 10.2215/CJN.10441015 27281698 PMC4934843

[34] Gupta J, Mitra N, Kanetsky PA, Devaney J, Wing MR, Reilly M, et al. Association between albuminuria, kidney function, and inflammatory biomarker profile in CKD in CRIC. Clin J Am Soc Nephrol [Internet]. 2012/09/27. 2012 Dec;7(12):1938–46. Available from: https://pubmed.ncbi.nlm.nih.gov/23024164. doi: 10.2215/CJN.03500412 23024164 PMC3513744

[35] Strober W, Waldmann TA. The role of the kidney in the metabolism of plasma proteins. Nephron. 1974;13(1):35–66. doi: 10.1159/000180368 4607245

[36] Argyropoulos CP, Chen SS, Ng Y-H, Roumelioti M-E, Shaffi K, Singh PP, et al. Rediscovering Beta-2 Microglobulin As a Biomarker across the Spectrum of Kidney Diseases. Front Med [Internet]. 2017 Jun 15;4:73. Available from: https://pubmed.ncbi.nlm.nih.gov/28664159. doi: 10.3389/fmed.2017.00073 PMC5471312 28664159

[37] Joseph Y, N. BE, C. MA, E. MR, J. WS, K. DR, et al. Impact of Kidney Function on the Blood Proteome and on Protein Cardiovascular Risk Biomarkers in Patients With Stable Coronary Heart Disease. J Am Heart Assoc [Internet]. 2020 Aug 4;9(15):e016463. Available from: 10.1161/JAHA.120.016463 32696702 PMC7792282

[38] Liu X, Foster MC, Tighiouart H, Anderson AH, Beck GJ, Contreras G, et al. Non-GFR Determinants of Low-Molecular-Weight Serum Protein Filtration Markers in CKD. Am J kidney Dis Off J Natl Kidney Found. 2016 Dec;68(6):892–900. doi: 10.1053/j.ajkd.2016.07.021 27663042 PMC5123901

[39] Christensson A, Ash JA, DeLisle RK, Gaspar FW, Ostroff R, Grubb A, et al. The Impact of the Glomerular Filtration Rate on the Human Plasma Proteome. PROTEOMICS–Clin Appl [Internet]. 2018 May 1;12(3):1700067. Available from: 10.1002/prca.201700067 29281176

[40] Luczak M, Formanowicz D, Pawliczak E, Wanic-Kossowska M, Wykretowicz A, Figlerowicz M. Chronic kidney disease-related atherosclerosis—proteomic studies of blood plasma. Proteome Sci [Internet]. 2011 May 13;9:25. Available from: https://pubmed.ncbi.nlm.nih.gov/21569504. doi: 10.1186/1477-5956-9-25 21569504 PMC3112376

[41] Jotwani V, Katz R, Ix JH, Gutiérrez OM, Bennett M, Parikh CR, et al. Urinary Biomarkers of Kidney Tubular Damage and Risk of Cardiovascular Disease and Mortality in Elders. Am J Kidney Dis [Internet]. 2018/03/27. 2018 Aug;72(2):205–13. Available from: https://pubmed.ncbi.nlm.nih.gov/29602632. doi: 10.1053/j.ajkd.2017.12.013 29602632 PMC6057830

[42] Vyssoulis GP, Tousoulis D, Antoniades C, Dimitrakopoulos S, Zervoudaki A, Stefanadis C. α-1 Microglobulin as a New Inflammatory Marker in Newly Diagnosed Hypertensive Patients:. Am J Hypertens [Internet]. 2007 Sep 1;20(9):1016–21. Available from: 10.1016/j.amjhyper.2007.01.010 17765145

[43] Volanakis JE, Barnum SR, Giddens M, Galla JH. Renal Filtration and Catabolism of Complement Protein D. N Engl J Med [Internet]. 1985 Feb 14;312(7):395–9. Available from: 10.1056/NEJM198502143120702 3844050

[44] Velho G, Ragot S, Mohammedi K, Gand E, Fraty M, Fumeron F, et al. Plasma Adrenomedullin and Allelic Variation in the ADM Gene and Kidney Disease in People With Type 2 Diabetes. Diabetes [Internet]. 2015 May 6;64(9):3262–72. Available from: 10.2337/db14-1852 25948679

[45] Fliser D, Kollerits B, Neyer U, Ankerst DP, Lhotta K, Lingenhel A, et al. Fibroblast Growth Factor 23 (FGF23) Predicts Progression of Chronic Kidney Disease: The Mild to Moderate Kidney Disease (MMKD) Study. J Am Soc Nephrol [Internet]. 2007 Sep 1;18(9):2600 LP– 2608. Available from: http://jasn.asnjournals.org/content/18/9/2600.abstract. doi: 10.1681/ASN.2006080936 17656479

[46] Dieplinger B, Mueller T, Kollerits B, Struck J, Ritz E, von Eckardstein A, et al. Pro-A-type natriuretic peptide and pro-adrenomedullin predict progression of chronic kidney disease: the MMKD Study. Kidney Int. 2009 Feb;75(4):408–14. doi: 10.1038/ki.2008.560 19052536

[47] Pena MJ, Heinzel A, Heinze G, Alkhalaf A, Bakker SJL, Nguyen TQ, et al. A panel of novel biomarkers representing different disease pathways improves prediction of renal function decline in type 2 diabetes. PLoS One [Internet]. 2015 May 14;10(5):e0120995–e0120995. Available from: https://pubmed.ncbi.nlm.nih.gov/25973922. doi: 10.1371/journal.pone.0120995 25973922 PMC4431870

[48] Matías-García PR, Wilson R, Guo Q, Zaghlool SB, Eales JM, Xu X, et al. Plasma Proteomics of Renal Function: A Transethnic Meta-Analysis and Mendelian Randomization Study. J Am Soc Nephrol [Internet]. 2021 Jul 1;32(7):1747 LP– 1763. Available from: http://jasn.asnjournals.org/content/32/7/1747.abstract. 10.1681/ASN.2020071070 PMC8425654 34135082

[49] Paunas FTI, Finne K, Leh S, Osman TA-H, Marti H-P, Berven F, et al. Characterization of glomerular extracellular matrix in IgA nephropathy by proteomic analysis of laser-captured microdissected glomeruli. BMC Nephrol [Internet]. 2019 Nov 14;20(1):410. Available from: https://pubmed.ncbi.nlm.nih.gov/31726998. doi: 10.1186/s12882-019-1598-1 31726998 PMC6854890

[50] Lin Z, Wang Z, Li G, Li B, Xie W, Xiang D. Fibulin-3 may improve vascular health through inhibition of MMP-2/9 and oxidative stress in spontaneously hypertensive rats. Mol Med Rep [Internet]. 2016/03/21. 2016 May;13(5):3805–12. Available from: https://pubmed.ncbi.nlm.nih.gov/27035767. doi: 10.3892/mmr.2016.5036 27035767 PMC4838143

[51] Fan Y, Fei Y, Zheng L, Wang J, Xiao W, Wen J, et al. Expression of Endothelial Cell Injury Marker Cd146 Correlates with Disease Severity and Predicts the Renal Outcomes in Patients with Diabetic Nephropathy. Cell Physiol Biochem [Internet]. 2018;48(1):63–74. Available from: https://www.karger.com/DOI/10.1159/000491663 30001528 10.1159/000491663

[52] Saito T, Saito O, Kawano T, Tamemoto H, Kusano E, Kawakami M, et al. Elevation of serum adiponectin and CD146 levels in diabetic nephropathy. Diabetes Res Clin Pract. 2007 Oct;78(1):85–92. doi: 10.1016/j.diabres.2007.02.014 17490776

[53] Bardin N, Moal V, Anfosso F, Daniel L, Brunet P, Sampol J, et al. Soluble CD146, a novel endothelial marker, is increased in physiopathological settings linked to endothelial junctional alteration. Thromb Haemost. 2003 Nov;90(5):915–20. doi: 10.1160/TH02-11-0285 14597988

[54] Wang N, Fan Y, Ni P, Wang F, Gao X, Xue Q, et al. High glucose effect on the role of CD146 in human proximal tubular epithelial cells in vitro. J Nephrol. 2008;21(6):931–40. 19034879

[55] Levin A, Stevens P, Bilous RW, Coresh J, Francisco A, Jong PE, et al. Kidney disease: Improving global outcomes (KDIGO) CKD work group. KDIGO 2012 clinical practice guideline for the evaluation and management of chronic kidney disease. Kidney Int Suppl. 2013 Jan 1;3:1–150.

